# Two conformations of the Tom20 preprotein receptor in the TOM holo complex

**DOI:** 10.1073/pnas.2301447120

**Published:** 2023-08-14

**Authors:** Pamela Ornelas, Thomas Bausewein, Janosch Martin, Nina Morgner, Stephan Nussberger, Werner Kühlbrandt

**Affiliations:** ^a^Department of Structural Biology, Max-Planck-Institute of Biophysics, Frankfurt 60438, Germany; ^b^Department of Structural Biology, Institute of Physical and Theoretical Chemistry, Goethe University of Frankfurt, Frankfurt 60439, Germany; ^c^Department of Biophysics, Institute of Biomaterials and Biomolecular Systems, University of Stuttgart, Stuttgart 70569, Germany

**Keywords:** cryoEM, translocation, TOM complex, Tom20

## Abstract

The TOM complex is the main entry point for precursor proteins (preproteins) into mitochondria. Preproteins containing targeting sequences are recognized by the TOM complex and imported into mitochondria. We have determined the structure of the TOM core complex from *Neurospora crassa* by single-particle electron cryomicroscopy at 3.3 Å resolution, showing its interaction with a bound preprotein at 4 Å resolution, and of the TOM holo complex including the Tom20 receptor at 6 to 7 Å resolution. TOM is a transmembrane complex consisting of two β-barrels, three receptor subunits, and three short transmembrane subunits. Tom20 has a transmembrane helix and a receptor domain on the cytoplasmic side. We propose that Tom20 acts as a dynamic gatekeeper, guiding preproteins into the pores of the TOM complex. We analyze the interactions of Tom20 with other TOM subunits, present insights into the structure of the TOM holo complex, and suggest a translocation mechanism.

## Significance

Most mitochondrial proteins are encoded in the cellular nucleus and produced as precursors in the cytoplasm. Precursor proteins are recognized and translocated into the inter-membrane space by the translocase of the outer mitochondrial membrane (TOM). We studied the conformation and composition of the TOM holo complex from *Neurospora crassa*, including the elusive receptors Tom20 and Tom70. We determined the structure of the complex under native conditions by cryoEM (electron cryomicroscopy), which shows the flexible gatekeeper, Tom20, in two different conformations. Furthermore, we present the structure of the complex with bound preprotein at a late stage of translocation. Overall, we offer insights into the dynamic process of presequence recognition and translocation into mitochondria, dependent on the cooperation of Tom20 and Tom22.

The translocase of the outer membrane (TOM) complex is an essential component of the outer membrane of mitochondria that acts as the main gate for protein import (1). Owing to the endosymbiotic origins of mitochondria, most of their proteins are produced in the cytosol as soluble precursors which are imported into the organelle (2, 3). Most of these proteins contain an N-terminal presequence that forms an amphipathic α-helix and acts as a mitochondrial targeting signal (MTS) (4, 5). Together with a diverse system of other import machineries, such as the sorting assembly machinery (SAM) in the outer membrane, the tanslocases of the inner mitochondrial membrane (TIM22 and TIM23) in the inner membrane, and multiple chaperones in the inter-membrane space (IMS) and matrix, the TOM complex ensures that these proteins reach their final destination within the mitochondrion (6).

The TOM core complex of *Neurospora crassa* (NcTOM) in the mitochondrial outer membrane is a dimer with 5 subunits per protomer: Tom40, Tom22, and the small Toms (sT) Tom5, Tom6, and Tom7 (7). It has a mass of 148 kDa (8) and dimensions of roughly 130 Å by 100 Å (9). The protein translocation pores are formed by two copies of Tom40, each a 19-strand β-barrel with helical termini. Between the pores, two copies of the α-helical Tom22 span the membrane, with its disordered N and C-terminal domains facing the cytosol and IMS. The pronounced negative charge of the Tom22 N terminus suggests that it interacts with the positive MTS of precursor proteins (preproteins) (10, 11). The small α-helical subunits Tom5, Tom6, and Tom7 are involved in complex assembly and are thought to play a role in stability and presequence recognition (12, 13).

In addition to the core subunits, the larger TOM holo complex contains the two receptor subunits Tom20 and Tom70 (14). Tom20 has a soluble core domain with 5 α-helices, including a tetratricopeptide repeat (TPR) at its C terminus and a transmembrane helix at its N terminus (15). Tom20 is thought to interact hydrophobically with matrix-targeted preproteins and has been suggested to cooperate with Tom22 in the early steps of translocation (15, 16). Tom70 consists of 26 α-helices in a large soluble domain forming 11 TPR motives that are connected by a disordered loop to a transmembrane helix (17, 18). Tom70 mostly recognizes carrier proteins and interacts with protein chaperones such as Hsp70 and Hsp90, but also cooperates with the mitochondrial import protein Mim1 in membrane protein biogenesis (19, 20).

The structure of NcTOM has been investigated in negative-stain (7) and by electron cryomicroscopy (cryoEM), which yielded a 6.8 Å map (9). CryoEM structures of the yeast and human complex have been published at around 20 Å (21) and, more recently, at 3 to 4 Å resolution (22–24). The TOM holo complex is a challenging target because Tom20 and Tom70 attach to the core complex only loosely (7, 25). Assembly, stoichiometry, and interaction of Tom20 and Tom70 with the core subunits remain largely unknown. Recently, the structure of a TOM dimer with the Tom20 core domain chemically cross-linked to Tom40 has been reported, suggesting the presence of two copies of Tom20 per TOM dimer (26). We now set out to determine the high-resolution structure of the NcTOM core complex with bound preprotein by single-particle cryoEM and to analyze the subunit composition of TOM holo complex through laser-induced liquid bead ion-desorption mass spectrometry (LILBID-MS). LILBID-MS is a native mass spectrometry technique that can examine intact as well as partially dissociated protein complexes to identify subunit interactions (27, 28).

We present a 3.3 Å structure of the NcTOM core complex and a structure of NcTOM interacting with the positive MTS of rat aldehyde dehydrogenase visible at a lower density threshold. Furthermore, we present two structures showing the TOM core complex interacting with the peripheral components of Tom20 at about 7 Å resolution. Our map indicates that Tom20 is flexible, taking on two distinct conformations, with Tom22 as a docking platform.

## Results

### Isolation of the TOM Holo Complex.

To investigate the structure of the TOM holo complex, we isolated mitochondria from *N. crassa* hyphae with recombinant Tom22 containing a hexahistidine tag (9). We solubilized outer membrane vesicles (OMVs) in glyco-diosgenin (GDN) and isolated the complex by affinity purification and size-exclusion chromatography. Gel electrophoresis indicated that peak fractions (*SI Appendix*, Fig. S1) contained all subunits of the TOM holo complex, including Tom20 and Tom70 (14). An additional band at around 30 kDa suggested the presence of the mitochondrial voltage-dependent anion channel (VDAC) (29), a common contaminant in TOM preparations.

### Native Mass Spectrometry of TOM.

**Fig. 1.**
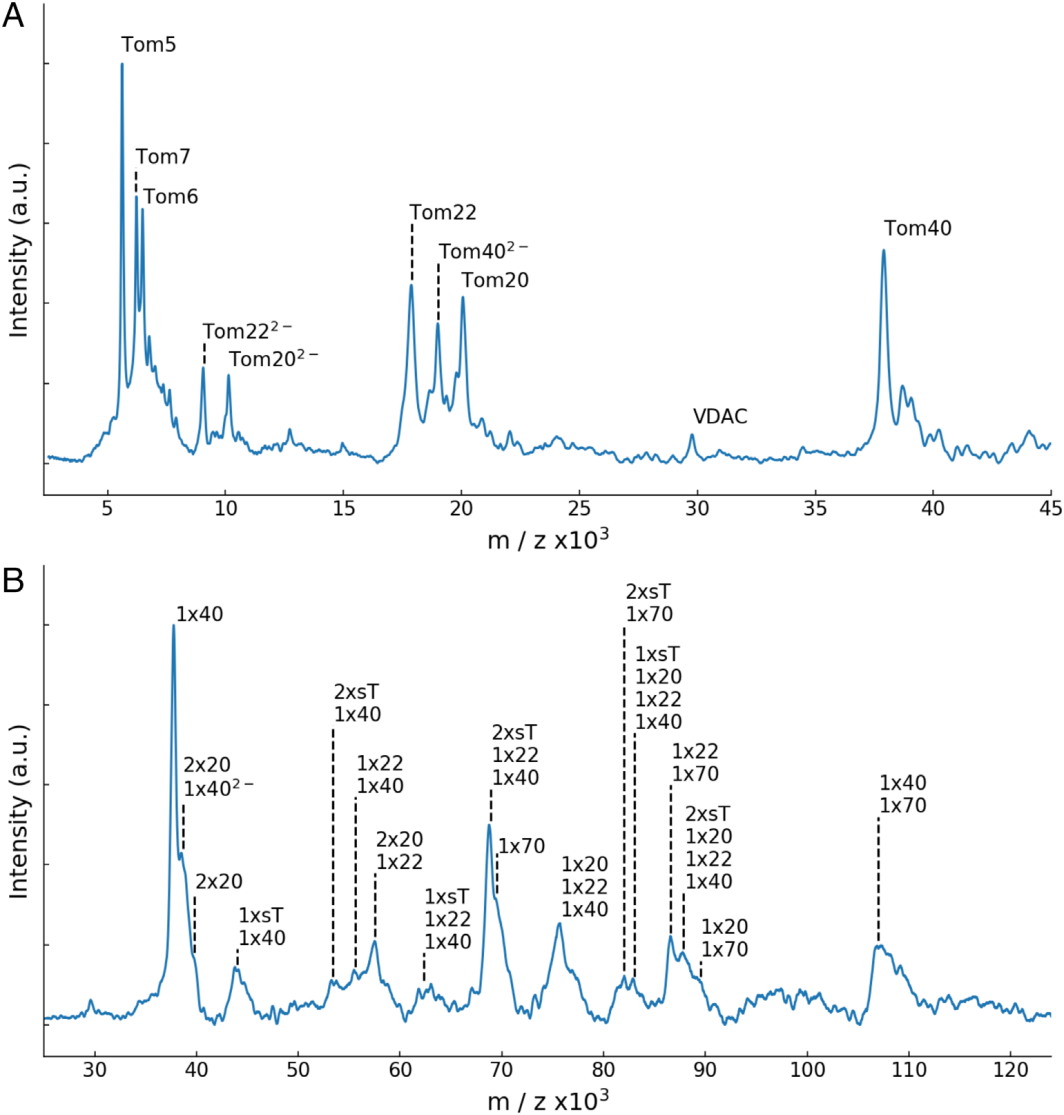
LILBID mass spectrometry of the TOM holo complex at high (*A*) and low (*B*) laser intensities. Peaks were assigned according to their predicted molecular weights (ExPASy). (*A*) In the lower mass region, individual dissociated subunits are visible under harsh laser conditions. Tom5, Tom6, and Tom7 appear as singly-charged entities, while Tom20, Tom22, and Tom40 carry one or two negative charges. A smaller peak was assigned to VDAC. (*B*) At reduced laser intensity, stable subcomplexes are visible in the mass range up to 125,000 m/z. Tom70 appears as a single molecule and forms subcomplexes with the small TOM subunits (sT), Tom20, Tom22, and Tom40. Tom20 forms subcomplexes with itself, with Tom22 and Tom40.

We investigated the composition of the TOM holo complex and subunit interactions by LILBID-MS. Fig. 1 shows spectra up to 125,000 m/z, indicating the different subunits and fragment subcomplexes of the holo complex at two different laser intensities. Monomeric forms of the core subunits appear at high laser intensities (8), as seen in Fig. 1*A*. In addition, we identified the peaks for singly and doubly-charged Tom20 at 20,100 m/z and 10,200 m/z respectively. The predicted molecular mass of Tom20 is 20.23 kDa. The peak at 29,800 m/z can be assigned to the VDAC contaminant.

At reduced laser intensity (Fig. 1*B*) we observed peaks indicating larger assemblies. We identified the individual subunit Tom70 at 69,300 m/z, closely matching its predicted mass of 69.34 kDa. Additional peaks were assigned to subcomplexes formed by core subunits and holo receptors, revealing, for example, a stable interaction of Tom70 with two sT, labeled as sT_2_Tom70_1_. Likewise, Tom70 interacts with the translocation pore forming a Tom40_1_Tom70_1_ subcomplex and with the receptor subunits forming the subcomplexes Tom20_1_Tom70_1_ and Tom22_1_Tom70_1_. We see evidence of a complex with Tom20_1_Tom22_1_Tom40_1_ stoichiometry, which contains also one or two sT, which might indicate a protomeric TOM holo assembly. This relates to the subcomplexes formed by Tom22, Tom40, and a variable number of sTs, as reported (8). Interestingly, we see Tom20_2_ dimers, also forming subcomplexes with other subunits, suggesting the presence of two copies of Tom20 per complex. Previously, the crystal structure of Tom20 with bound presequence (PDB 2V1S) was reported to be a dimer, although dimer formation was reported as biologically irrelevant, due to the nature of the residues involved in the interaction (30). Peak assignments based on subunit mass can be found in *SI Appendix*, Table S1.

### Structure of the Dimeric TOM Core Complex.

**Fig. 2.**
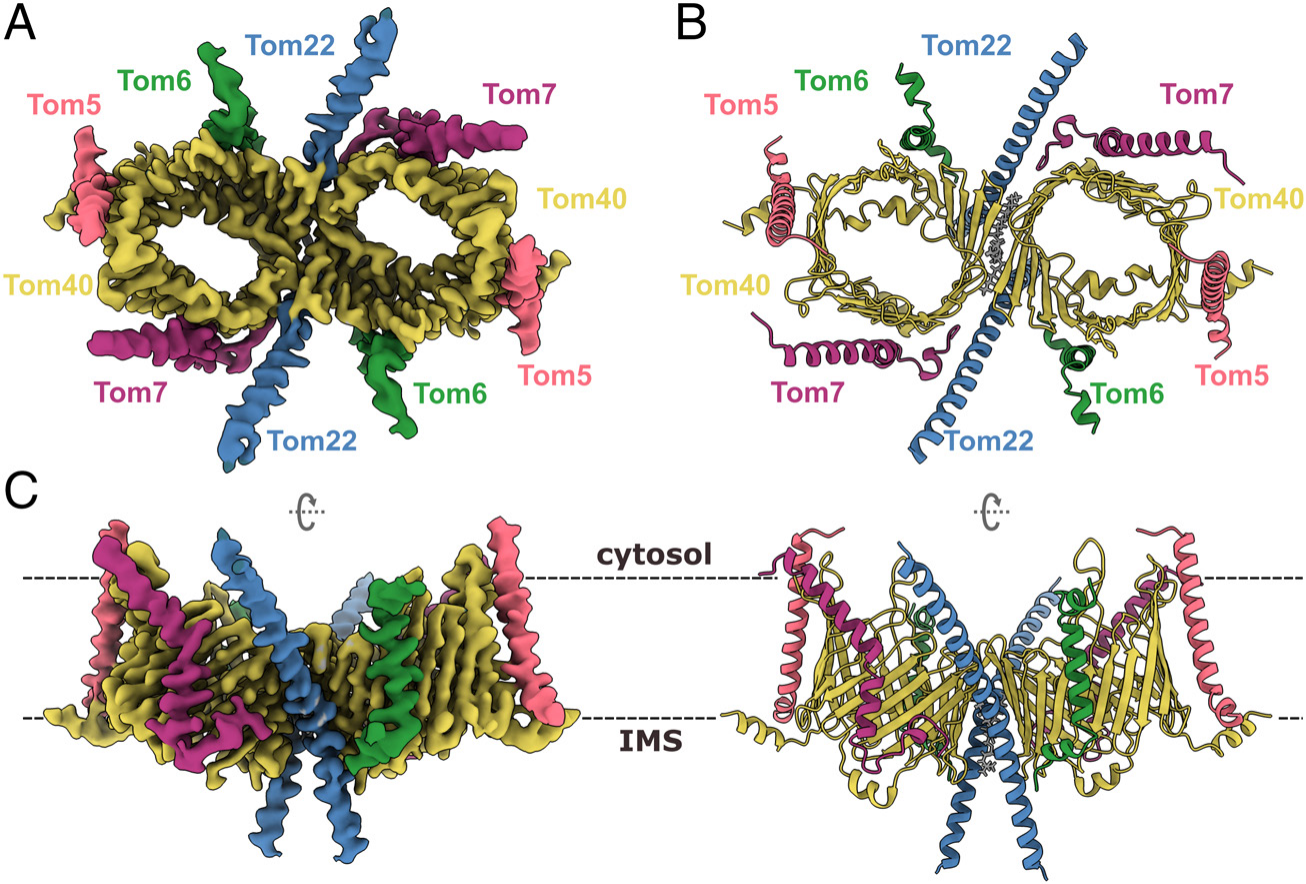
CryoEM map and model of the TOM core complex from *N. crassa*. Map and model are colored by subunit. Tom40, yellow; Tom22, blue; Tom5, Tom6, and Tom7 are pink, green, and purple, respectively. (*A*) TOM complex dimer at 3.32 Å resolution seen from the cytosol. (*B*) Cartoon representation of the atomic model, including a lipid molecule between the two pores seen from the cytosol. (*C*) Side view of the map and model from the outer mitochondrial membrane. The approximate position of the lipid bilayer is indicated by dashed lines.

To study the TOM translocation mechanism, we incubated the purified holo complex with a synthetic peptide containing the presequence of rat aldehyde dehydrogenase (pALDH). Rat pALDH is a common target in the study of mitochondrial translocation and has been observed to interact with Tom20 (15, 30, 31). We then plunge-froze the mixture and performed single-particle analysis of the complex in GDN, which resulted in a 3.3 Å resolution map of the TOM core complex to which we applied C2 symmetry during the final reconstruction step (Fig. 2*A* and *SI Appendix*, Fig. S2). We identified the 5 core subunits of NcTOM based on our published 6.8 Å structure of the dimeric core complex (9). Our high-resolution TOM core map indicates two copies of the Tom40 barrel, the helical Tom22, Tom5, Tom6, and Tom7 subunits, but it lacks the receptors Tom20 and Tom70 and the presequence.

We built an atomic model (Fig. 2*B* and *SI Appendix*, Fig. S3), starting from a prediction model generated with the program AlphaFold-Multimer. Our map contains clear densities for the 19 individual β-strands of the Tom40 barrel and the loops connecting them (*SI Appendix*, Fig. S4). The longest loop, joining strands 14 and 15, is visible at a lower density threshold, indicating that it is flexible. As observed in our previous model of Tom40 (PDB 5O8O), the N terminus starts with two short helices (α1 and α2), and the C terminus ends in one helix (α3). Helix α1 starts outside the pore and interacts with Tom5 at the IMS. After an unstructured but highly conserved stretch, it turns into helix α2 inside the Tom40 pore, interacting with β-strands 11 to 16. Internal short helices such as α2 are common features of membrane-embedded β-barrels, and their mutation or deletion can lead to structural reshaping and destabilizing of the barrels (32, 33). At the end of β-strand 19, α3 extends into the IMS and folds back into the pore to interact with β-strand 4. Due to flexibility, α3 is only visible at a lower density threshold, while its C-terminal residues are well ordered. At the point of contact between the monomers, we see interactions between β-strands 19, 1, and 2 of the two Tom40 barrels.

**Fig. 3.**
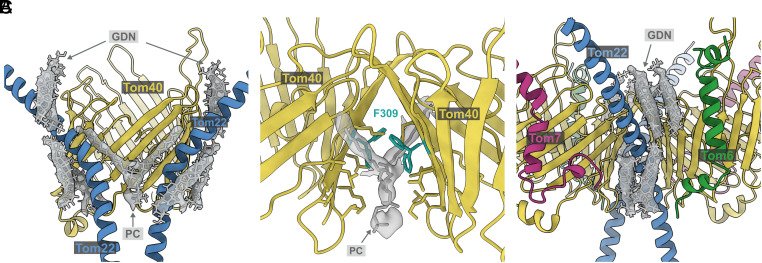
Lipid and detergent densities near Tom22 and Tom40. (*A*) One PC and eight molecules of GDN interact with the dimeric TOM core complex. (*B*) Close-up of PC at the dimer interface, where we see two rotamer conformations for F309 in Tom40. (*C*) Four molecules of GDN were identified around each monomer of the complex.

As we found previously, the two TOM core protomers are tilted relative to each other at an angle of ~20° (9). In the space between the protomers, we identified a phospholipid that interacts with Tom40 and Tom22 (Fig. 3*A*). The lipid is in contact with strands 17, 18, and 19 of both Tom40s and appears to interact hydrophobically with the F309 sidechain, for which we see two rotamer conformations (Fig. 3*B*). The map shows another four elongated, bulky densities on each side of the dimer, in close contact with Tom40 and Tom22. We assigned these densities that each span half of the membrane to GDN detergent molecules used for solubilization (Fig. 3*C*). Nearly 70% of the mitochondrial outer membrane is accounted for by PC and phosphatidylethanolamine (34); we propose that the remaining unmodeled elongated densities around the dimer in the map correspond to these lipids.

Our model also contains two copies of the Tom22 transmembrane helix. The hydrophilic N and C termini of Tom22, which interact with the presequence and other subunits (35), are not resolved and evidently disordered (Fig. 2*C*). Similarly, the flexible N termini of Tom5 and Tom6 are not visible, while their transmembrane domains are clearly helical. In our map, Tom7 is mostly complete, embedded in the membrane and displaying its characteristic Z shape. Our assignment of NcTOM subunits matches our earlier 6.8 Å map (EMD-3761), except for the IMS domain of Tom6, which appeared to be longer in our previous map (*SI Appendix*, Fig. S5), perhaps due to anisotropic resolution.

### Preprotein Translocation.

**Fig. 4.**
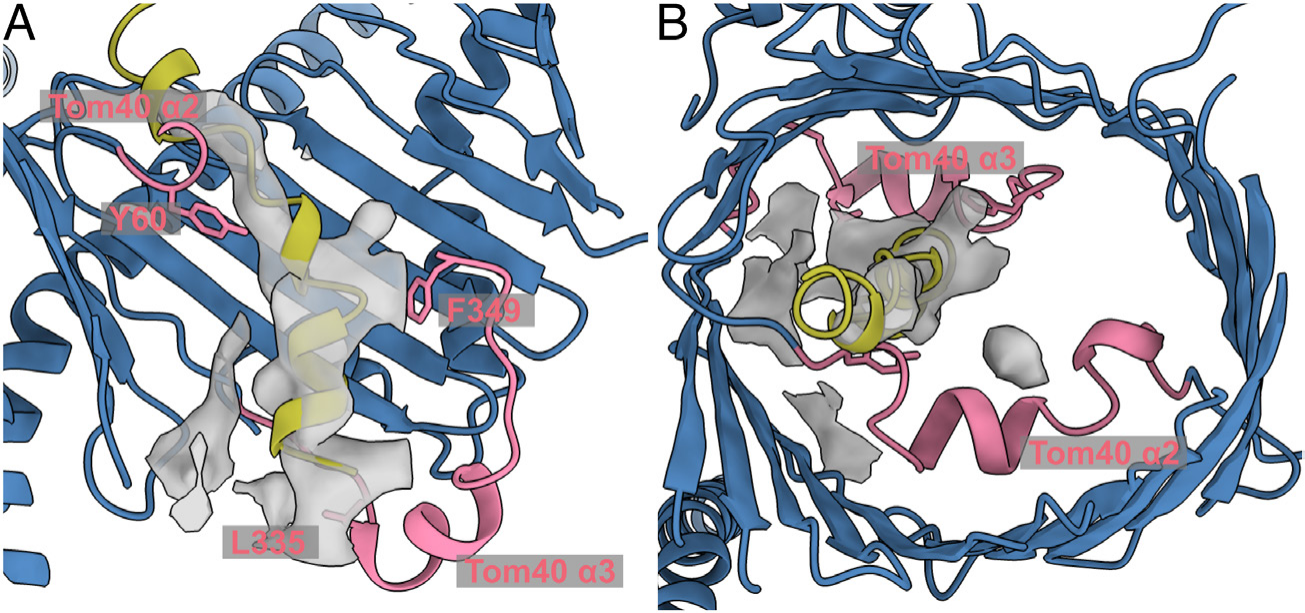
Difference density map indicating preprotein bound to Tom40. Transversal sections through the difference map and fitted model indicated a presequence density inside the Tom40 translocation pore. The pALDH structure generated with AlphaFold is shown in yellow, the 4 Å map of the TOM core complex in gray, the TOM core model in blue, and the two inner helices of Tom40 in pink. (*A*) Side view of the inner Tom40 pore showing Y60, F349, and L335. The density inside the pore spans from the cytoplasmic side of the complex toward the IMS, interacting on its way with α2 and α3. (*B*) View of the pore from the cytosol.

Refinement of the same single-particle dataset with limited alignment resolution yielded a C2-symmetrical 4 Å map, in which each of the translocation pores contained an elongated density crossing the Tom40 barrel (Fig. 4), visible at lower thresholds (*SI Appendix*, Fig. S6). A difference density map between this map and a map generated from the NcTOM core model showed individual densities that appear to belong to the pALDH preprotein captured in translocation. The map in Fig. 4 shows the interaction of the translocated preprotein, superimposed on our TOM core C2 model. Extending from the cytosol into the IMS, the density makes contact with regions rich in hydrophobic residues, closely approaching Y60 in α2, and L335 and F349 at the end of α3. This suggests a possible involvement of Y60 and L335 in presequence recognition and translocation.

### Interaction of Tom20 with the TOM Core Complex.

We extracted more information on the cytosolic domains of Tom20 and Tom22 from the same dataset at lower resolution. After two rounds of three-dimensional (3D) classification and refinement, we obtained two maps that we assign to two different positions of Tom20, referred to as P_1_ and P_2_, at 6.7 Å and 6.6 Å resolution, respectively (*SI Appendix*, Figs. S2 and S7). The rod-shaped density of an α-helix protrudes from the edge of the micelle and appears to be suspended over the pores. At its end, a globular domain becomes visible at a lower density threshold.

**Fig. 5.**
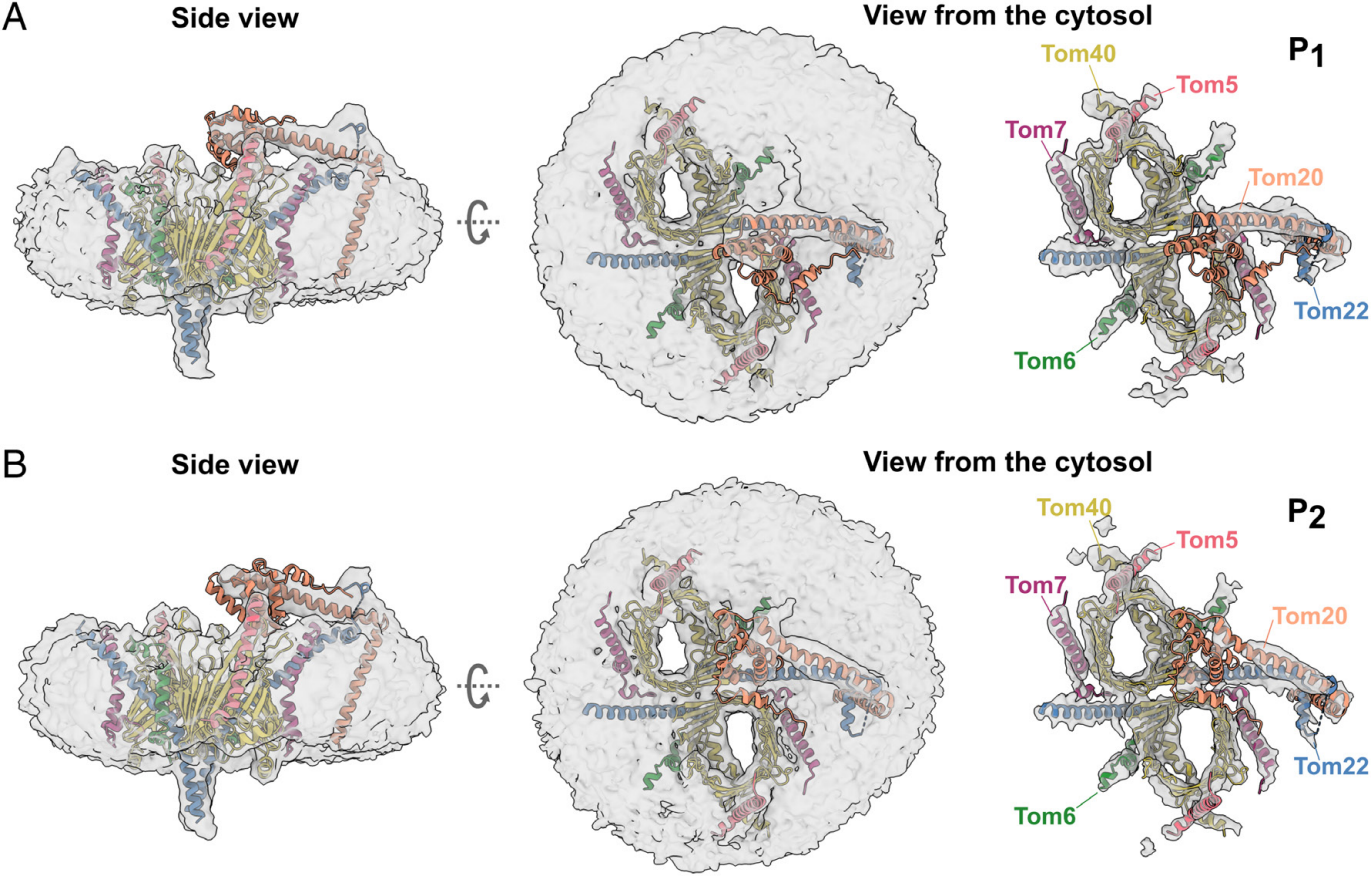
Tom20 assumes two discrete positions on the cytosolic side. Fit of an AlphaFold model of Tom20 to the cryoEM map of the TOM holo complex at 6.7 Å (*A*) and 6.6 Å (*B*), superimposed with the core model. The cytosolic domain of Tom20 (orange) assumes two distinct positions (P_1_ and P_2_) aligned with Tom22 at the cytosol membrane in the center of the TOM core dimer or closer to one pore, where it appears to interact with Tom6.

We fitted the AlphaFold prediction models for Tom20 and Tom22 into both maps as rigid bodies (*SI Appendix*, Fig. S8). As shown in Fig. 5, we observe that one Tom22 helix bends further to the side of the core complex on the cytosolic side, as in the *Homo sapiens* core complex (24), and stretches across the asymmetric micelle. The transmembrane helix of Tom20 emerges from the micelle and interacts with Tom22 at the membrane surface. A helix, with its connected receptor domain, appears to hover above the translocation pores, roughly parallel to the membrane plane. At the interface, part of the N terminus of Tom22 wraps around Tom20, connecting it to the docking site.

The two maps differ in the position of Tom20 on the cytoplasmic side of the complex. In Fig. 5*A*, Tom20 is situated right on top and in between the translocation pores (P_1_), while in Fig. 5*B*, Tom20 leans toward one of the pores within close range of Tom6 and the loop connecting Tom40 strands 14 and 15 (P_2_). This might indicate that the receptor domain of Tom20 serves both pores as a flexible gatekeeper and shuttles between them.

## Discussion

We used cryoEM and LILBID-MS to gain insight into the structure and translocation mechanism of the TOM complex. Our TOM core map shows a conserved structure, in close agreement to the previously published cryoEM structures of *N. crassa*, yeast, and human TOM. However, specific differences between species are evident (*SI Appendix*, Fig. S9). The C terminus of NcTom40 is a flexible helix that extends into the IMS and folds back into the translocation pore, while in the human complex, this helix is replaced by a longer Tom7 C terminus. In yeast, a corresponding helix is instead oriented toward Tom22 (36). These differences are visible in the IMS exit pathway and would affect the interaction of the complex with preproteins. Similarly, the Tom40 loop between strands 14 and 15 is considerably longer in *N. crassa* than in the human complex, which might influence preprotein insertion into the pore. We confirm the presence of a phospholipid at the interface between the two copies of Tom40, which might serve to maintain the tilt angle between the two Tom40 barrels (22). The four elongated strong densities around each Tom22 that we assign to GDN molecules are bound to be occupied by lipids in the membrane.

Our 4 Å map with limited alignment resolution contains a density inside the Tom40 pores, consistent with earlier research suggesting that Tom40 uses a combination of acidic and hydrophobic patches to translocate the presequence toward the IMS (23, 37, 38). Cross-linking studies have demonstrated presequence binding to the cytoplasmic side of Tom40 (39). However, our density appears in the center of the pore close to the IMS exit site, most likely illustrating a late stage of the translocation process (*SI Appendix*, Fig. S6). This interaction would correspond to a trans-binding site within Tom40, supported by Tom7 and Tom22 (38, 40).

**Fig. 6.**
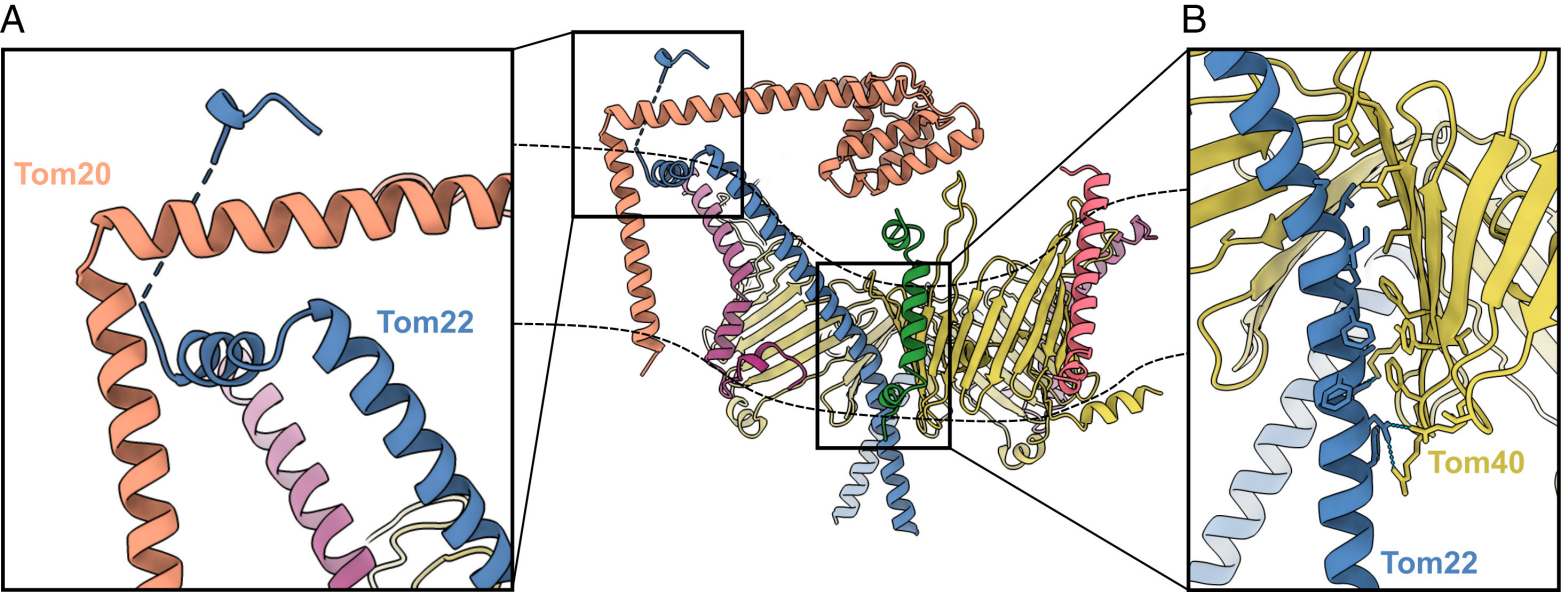
Interactions of Tom20, Tom22, and Tom40. Proposed interactions between Tom20 in orange, Tom22 in blue, and Tom40 in yellow, based on our models of the TOM core and holo complex. The position of the outer mitochondrial membrane is indicated by dashed lines. (*A*) Rigid-body-fitted model of Tom20 docked to Tom22. Tom20 is held in place by the N terminus of Tom22 wrapped around it. (*B*) Tom22 and Tom40 are held together in the membrane by hydrophobic and electrostatic contacts.

Our results shed new light on the function of the receptor Tom20. In contrast to a recent report on the human TOM complex (26), we neither cross-linked Tom20 to the core complex, nor did we impose twofold symmetry on our TOM holo maps. Our maps therefore represent the structure of the native holo complex which is asymmetric (Fig. 5), indicating that it contains only one copy of Tom20. Our LILBID-MS results show multiple subcomplexes composed of Tom20, Tom22, and Tom40 with different stoichiometries (Fig. 1*B*). This is consistent with a flexible cytosolic domain of Tom20, capable of taking on different conformations in its interactions with the TOM core complex. Our maps suggest a strong interaction of the acidic patch of the N terminus of Tom22 with a positively charged patch in the Tom20 helix (*SI Appendix*, Fig. S10), confirming that Tom22 is a docking point required for optimizing the receptor function of Tom20 (16, 31, 35). Based on our models, we propose that Tom20 docks to the Tom22 helix at the cytosolic membrane surface, while Tom22 and Tom40 bind strongly to each other through sidechain-specific hydrophobic and electrostatic contacts (Fig. 6).

**Fig. 7.**
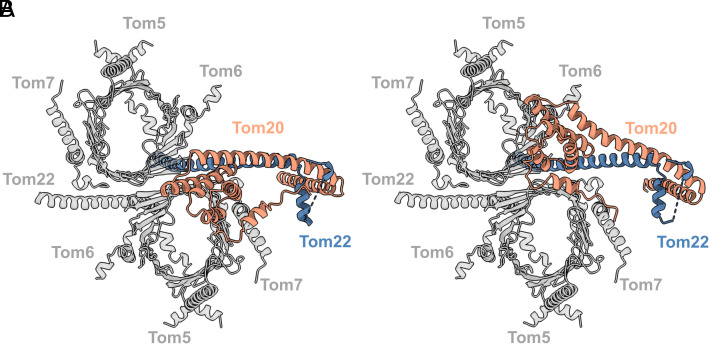
Model of the TOM complex including Tom20. Cartoon representation of Tom20 (orange) in two different positions as seen from the cytosol. Tom20 docks to Tom22 (blue), interacting with the TOM core complex (gray). In P_1_ (*A*), Tom20 assumes a central position between the two pores, while in P_2_ (*B*), Tom20 approaches one pore and interacts with Tom40 near Tom6.

We propose that in the native holo complex Tom20 takes on two alternative positions. In position P_1_, Tom20 lies parallel to the membrane, roughly aligned to Tom22, within close range of both translocation pores (Fig. 7*A*). In position P_2_, the Tom20 receptor domain approaches one of the pores and interacts with Tom40 and perhaps Tom6 (Fig. 7*B*). This second position agrees with cross-linking studies that indicate the interaction of the longest Tom40 loop between strands 14 and 15 with Tom20 (39), and the LILBID-MS subcomplexes formed by Tom20, Tom22, Tom40, and at least one small TOM subunit (Fig. 1*B*). Moreover, position P_2_ is consistent with the model predicted by AlphaFold for the Tom20_1_Tom22_1_Tom40_1_ subcomplex (*SI Appendix*, Fig. S8*A*). Additionally, other cross-linking studies have indicated the interaction of a preprotein with Tom6 in the early stages of translocation (41). Taking all this into consideration, we suggest that upon contact with the preprotein, Tom20 approaches Tom6, interacts with Tom40, and inserts the preprotein into the pore, initiating the translocation process.

Our nonsymmetrized map indicates one copy of Tom20 in the complex, although our LILBID-MS spectra suggest that a fraction of TOM subcomplexes can contain two copies of Tom20 (Fig. 1*B*). However, a minor population of TOM complexes may not be detected as a separate class in image processing. We nevertheless attempted to fit two copies of Tom20 in its two different conformations into our TOM core model but found that the two receptor domains would clash on the cytoplasmic side. A steric clash would make it unlikely that two copies of Tom20s can coexist simultaneously in one TOM dimer (*SI Appendix*, Fig. S11).

We see a peak corresponding to Tom70 in our LILBID-MS spectra (Fig. 1*B*), as well as subcomplexes formed by Tom70 in association with Tom20, Tom22, Tom40, and up to two small subunits, but we are not able to assign it to any particular density region. The interaction between Tom20 and Tom70 has been reported before in vitro in other organisms by cross-linking (42). However, a stable interaction might depend on Tom70 binding a preprotein. More work is required to establish the position of Tom70 relative to the core complex and to fully understand its function.

In conclusion, we propose a translocation pathway for the TOM core complex that includes a flexible Tom20 in its two observed positions (Movie S1). We propose that Tom22 not only acts as a preprotein receptor but serves as a docking platform for Tom20, stabilizing Tom20 through electrostatic interactions at its N terminus. Apparently, Tom20 can reach both pores from position P_1_ (Fig. 7*A*). We propose that upon contact with a presequence, Tom20 changes position to P_2_ (Fig. 7*B*), hovering above one pore where it interacts with the Tom40 loop between strands 14 and 15. In this way, Tom20 would deliver the preprotein to Tom40, which in turn translocates the preproteins by means of acidic and hydrophobic interactions.

## Materials and Methods

### Growth of *N. crassa* and Preparation of Mitochondrial OMVs.

*N. crassa* (strain GR-107) containing a hexahistidinyl-tagged form of Tom22 was cultured and mitochondria were isolated as described (43). Briefly, ~1.5 kg wet weight of hyphae was homogenized in 250 mM sucrose, 2 mM Ethylenediaminetetraacetic acid (EDTA), 20 mM Tris pH 8.5, and 1 mM phenylmethylsulfonyl fluoride (PMSF) in a Waring mixer at 4 °C. Approximately 1.5 kg of silica sand was added, and cell walls were broken by passing the suspension through a corundum mill. Cellular residues were pelleted and discarded in two centrifugation steps (4,000 × g) for 5 min at 4 °C. Mitochondria were sedimented in 250 mM sucrose, 2 mM EDTA, 20 mM Tris pH 8.5, and 1 mM PMSF at 17,000 g for 80 min. This step was repeated to improve purity. The isolated mitochondria were suspended in 250 mM sucrose, 20 mM Tris pH 8.5, and 1 mM PMSF with a final protein concentration of 50 mg/ml. Mitochondrial membranes were then separated from soluble matrix and IMS proteins by centrifugation at 17,700 g after mitochondria were swollen at a protein concentration of 2 mg/ml in 5 mM Tris pH 8.5, 1 mM EDTA, and 1 mM PMSF. To obtain the outer membranes of the mitochondria, the membrane pellets were resuspended in the same buffer and homogenized in an automated glass-Teflon douncer for 60 min at 4 °C to separate outer and inner membranes.

OMVs were isolated by sedimentation and flotation centrifugation of the homogenate in sucrose step gradients in 20 mM Tris pH 8.5, 1 mM EDTA, and 1 mM PMSF as previously described (14). The isolated outer membranes were diluted threefold with 50 mM Tris pH 8.5, sedimented by centrifugation at 250,000 g, resuspended in 50 mM Tris pH 8.5 with a protein concentration of ~1 mg/ml, and used directly to isolate the TOM holo complex.

### Purification of the TOM Holo Complex.

The TOM holo complex was isolated from OMVs in solubilization buffer (20% glycerol, 10 mM 3-(N-morpholino)propanesulfonic acid (MOPS) pH 7.0, 50 mM potassium acetate, 50 mM imidazole, 1 mM PMSF, and 1% GDN) at a protein concentration of 1 mg/ml. After incubation for 1 h at 4 °C, the lysate was centrifuged at 13,000 g for 20 min. The clarified extract was loaded onto a nickel-nitrilotriacetic acid column. Nonspecifically bound proteins were washed off using 10 mM MOPS pH 7.0, 50 mM potassium acetate, 50 mM imidazole, 1 mM PMSF, and 0.02% GDN). The complex was eluted with 300 mM imidazole in the same buffer and concentrated (AmiconUltra 100 kDa cutoff). The purity of the eluted fractions was assessed by sodium dodecyl sulfate polyacrylamide gel electrophoresis (SDS-PAGE) and Coomassie Brilliant Blue staining.

For LILBID-MS, we further purified TOM holo complex using a Superdex 200 Increase size exclusion column (Cytiva) equilibrated with 10 mM Tris pH 7.0, 15 mM ammonium acetate, and 0.02% GDN. After SDS-PAGE, the peak fractions containing the TOM holo complex were pooled and concentrated to 4 mg/ml (AmiconUltra 100 kDa cutoff). For CryoEM, the complex was incubated for 1 h with excess pALDH at a 1:8 ratio (44). The mix was loaded onto a Superdex 200 Increase size exclusion column (Cytiva) equilibrated with 50 mM KPO_4_ pH 8.0, 50 mM KCl, 1 mM EDTA, 1 mM tris(2-carboxyethyl)phosphine (TCEP), and 0.02% GDN. Fractions were assessed by SDS-PAGE and Coomassie Brilliant Blue staining.

### Laser-Induced Liquid Bead Ion Desorption Mass Spectrometry.

For LILBID-MS analysis, ions were generated with an IR laser from 50 µm microdroplets containing the proteins of interest (45). Microdroplets were produced by a commercially available piezo-driven droplet generator (MD-K-130; microdrop Technologies GmbH). The IR laser operated at the absorption wavelength of water (2.94 µm) and droplets were produced and irradiated at a frequency of 10 Hz. The IR-laser power was varied in a range of 10 mJ to 23 mJ. Ions were detected with a home-built time-of-flight analyzer, operating at a vacuum of 10^−6^ mbar. Each measurement was performed in negative ion mode with a sample volume of 4 µl. All shown mass spectra were normalized to 1 and represent averaged signals of 1,000 droplets. Spectra were analyzed, and data were processed with Massign (46). Peaks were assigned on the basis of predicted average molecular mass of the individual subunits, calculated using ExPASy (47) (see *SI Appendix*, Table S1 for details).

### Preprotein Preparation.

The precursor peptide pALDH was synthesized by GenScript, resuspended in H_2_O upon delivery, aliquoted, and frozen until used. The peptide consisted of the first 19 amino acids of the MTS of rat aldehyde dehydrogenase plus a StrepII-tag joined by a linker to its C terminus. The precursor peptide sequence including the linker was MLRAALSTARRGPRLSRLLSGGGSWSHPQFEK.

### CryoEM Specimen Preparation and Data Acquisition.

A TOM holo complex peak fraction containing ~2 mg/ml protein was used for cryoEM. Roughly 3 µl was applied to a glow-discharged C-Flat 1.2/1.3 Cu grid, blotted for 3 s at 100% humidity at 4 °C, and flash-frozen in a Vitrobot Mark IV (FEI). Images were recorded at 300 kV using a Titan Krios electron microscope (ThermoFisher Scientific) equipped with a Gatan K3 camera in counting mode and a Gatan BioQuantum energy filter. Movies were collected with aberration-free image shift and hole clustering in EPU (ThermoFisher Scientific). Dose-fractionated movies were acquired with 3 s exposure at a 105,000× nominal magnification, resulting in a pixel size of 0.83 Å. The total accumulated dose was 55 e^−^/A^2^. Image defocus was in the range of −1.2 to −3.0 µm.

### Single-Particle Analysis of the TOM Core Complex.

Images were processed using Relion-4.0 (48). Movies were motion-corrected using MotionCor2 (49), and CTF parameters were initially estimated using CTFFIND-4 (50), both as implemented in Relion. A particle-picking model was manually built using crYOLO (51) and subsequently applied to the entire dataset. After extraction, two-dimensional (2D) classification was used to discard artefacts, and a set of the 620,000 best particle images was separated by 3D classification. The initial model used for classification was obtained from a preliminary cryoSPARC Live ab initio classification. Initial 3D refinement in Relion reached a resolution of 4.6 Å, or 4.2 Å after Bayesian polishing. The polished particles were imported into cryoSPARC v3 (52), where nonuniform refinement produced a map at 3.6 Å resolution. After a round of 3D classification without alignment, 304,000 particles were subjected to nonuniform refinement with imposed C2 symmetry to 3.37 Å resolution. Local refinement in CryoSPARC with a mask around the entire core complex improved the resolution to 3.32 Å, as assessed by the gold standard Fourier shell correlation (FSC) 0.143 criterion. Local resolution was determined by cryoSPARC (see *SI Appendix*, Fig. S2 and Table S2 for details). The same particles were submitted to a nonuniform, global refinement with a 10 Å maximum align resolution limit and imposed C2 symmetry in cryoSPARC. The refined map had a resolution of 4 Å according to the gold standard FSC and was used to study the preprotein-bound TOM core complex. The presequence density was identified by subtraction of a map generated from the TOM core model using UCSF ChimeraX (53) (*SI Appendix*, Fig. S6). The detergent micelle was deleted from the difference map using the volume eraser.

### Single-Particle Analysis of the TOM Core + Tom20 Complex.

Polished particles were further processed in Relion. A mask covering the area assigned to Tom20 was created in UCSF ChimeraX. Following masked 3D classification without alignment (K = 5, T = 10), two classes with distinct Tom20 conformations were selected, with 140,000 and 120,000 particles each, and independently refined without enforced symmetry to 6.6 Å or 6.7 Å resolution, respectively (see *SI Appendix*, Figs. S2 and S7 for details).

### Model Building.

Atomic model building of the TOM core complex was based on the AlphaFold-Multimer (54) prediction of the core dimer, then fitted into the refined map using Coot (55) and ISOLDE (56) within UCSF ChimeraX. Additional real-space refinement was performed in Phenix (57). Phosphatidylcholine (PC) and diosgenin were fitted to the map. The structures of the pALDH construct and the oligomer formed by Tom20, Tom22, and Tom40 were predicted by AlphaFold-Multimer. The preprotein-bound TOM core complex was rigid-body-fitted with UCSF ChimeraX. The TOM core plus Tom20 model was based on the AlphaFold (58) predictions of Tom20 and Tom22 (*SI Appendix*, Fig. S8). For each conformation, Tom20 and Tom22 were rigid-body-fitted to the map, relaxed using Coot and then merged into the TOM core model (see *SI Appendix*, Table S2 for more information).

## Supplementary Material

Appendix 01 (PDF)

Movie S1. Morph of TOM core + Tom20 models changing between positions P_1_ and P_2_. In P_1_, Tom20 (orange) is in a central position, docked on Tom22 (blue), between the two pores of the dimer (gray). In P_2_, Tom20 approaches the pore close to Tom6.

## Data Availability

The cryoEM map and atomic model of the TOM core complex have been deposited in the Protein Data Bank and in the Electron Microscopy Data Bank with accession codes PDB 8B4I (59) and EMD-15849 (60). The cryoEM maps of the two conformations of the TOM complex with Tom20 have been deposited in the Electron Microscopy Data Bank with accession codes EMD-15850 (61) and EMD-15856 (62). All data needed to evaluate the conclusions are present in the paper and/or supporting information.
