# Supplementary material for: Two conformations of the Tom20 preprotein receptor in the TOM holo complex
Source: Proc Natl Acad Sci U S A. 2023 Aug 14;120(34):e2301447120. doi: 10.1073/pnas.2301447120 (PMC10450662; doi:10.1073/pnas.2301447120)
Supplement: Supplementary file 1 — Appendix 01 (PDF) [file pnas.2301447120.sapp.pdf]

PNAS

Supporting Information for

Two conformations of the Tom20 preprotein receptor in the TOM holo complex.

Pamela Ornelas[1], Thomas Bausewein[1†], Janosch Martin[2], Nina Morgner[2], Stephan Nussberger[3], Werner Kühlbrandt[1*]

[1] Max-Planck-Institute of Biophysics, Frankfurt, Germany, 60438
[2] Institute of Physical and Theoretical Chemistry, Goethe University of Frankfurt, Frankfurt, Germany, 60439
[3] Department of Biophysics, Institute of Biomaterials and Biomolecular Systems, University of Stuttgart, Stuttgart, Germany, 70569
[†] Present address: Vironova BioAnalytics AB, Stockholm, Sweden, 113 30

*Corresponding author: Werner Kühlbrandt
Email: werner.kuehlbrandt@biophys.mpg.de
Address: Max-von-Laue-Straße 3, 60438 Frankfurt am Main

This PDF file includes:

Figures S1 to S11
Tables S1 to S2
Legend for Movie S1

Other supporting materials for this manuscript include the following:

Movie S1

Supplementary Figures and Tables

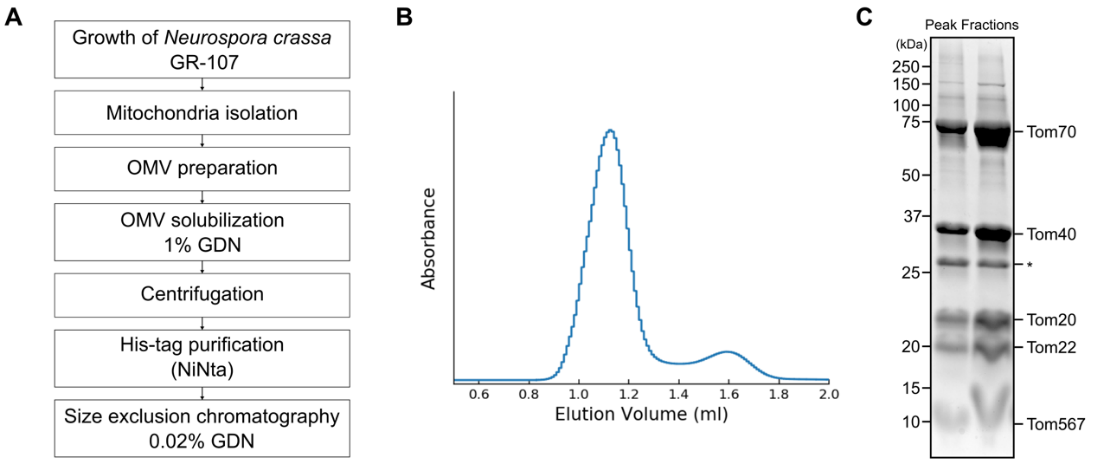

Fig. S1.

Purification of TOM holo complex from outer membrane vesicles. (**A**) TOM holo purification steps. (**B**) Size-exclusion chromatography profile of TOM holo complex in GDN using the Superdex 200 Increase column. (**C**) Coomassie-stained SDS-PAGE of the main peak after size-exclusion chromatography. The asterisk indicates the mitochondrial voltage-dependent anion channel (VDAC).

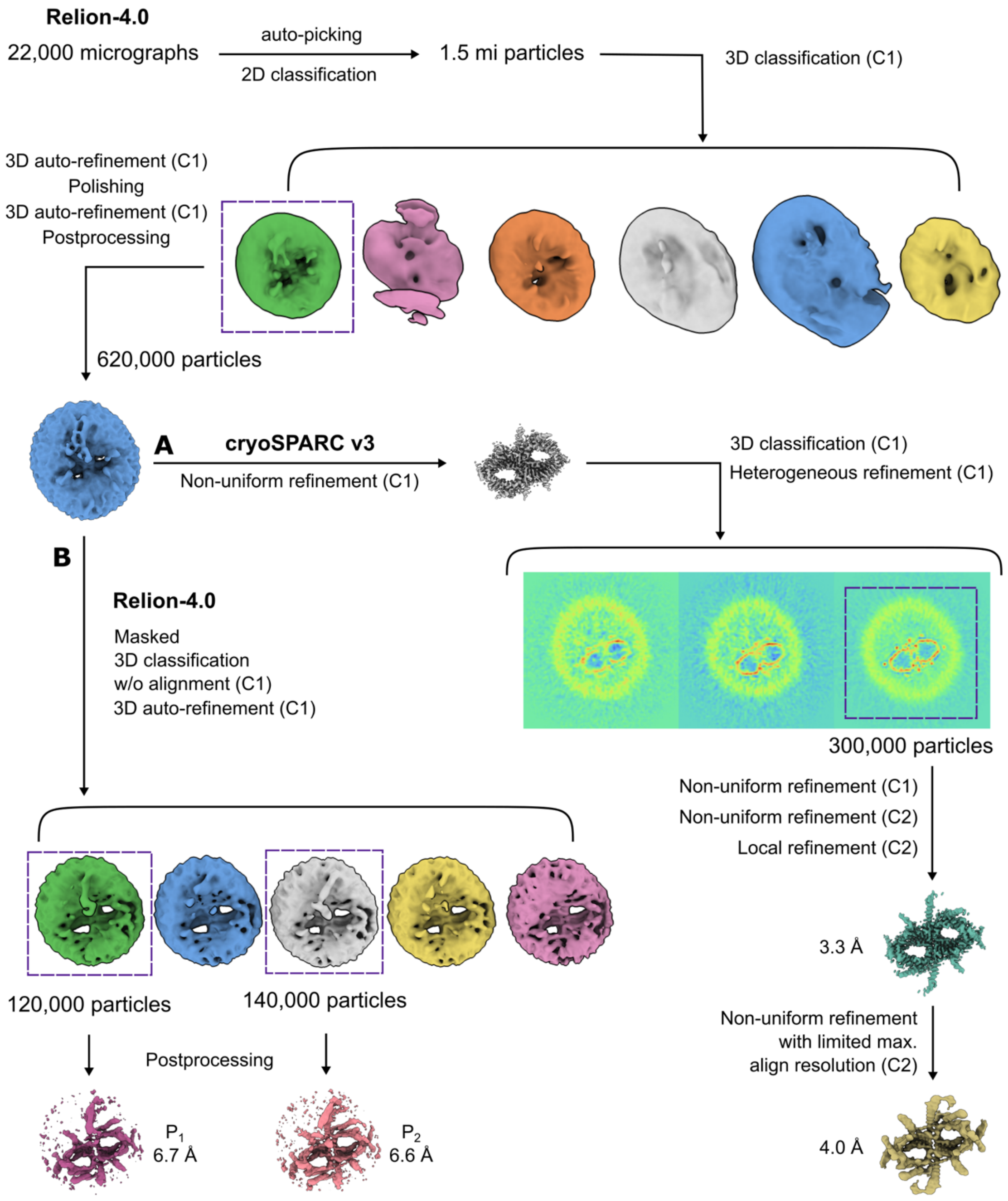

Fig. S2.

Single-particle cryoEM analysis of TOM complex structure. The workflow includes the steps leading to: (**A**) The 3.3 Å resolution map of TOM core complex and the 4 Å resolution map of the presequence-bound TOM core complex in cryoSPARC v3. (**B**) Maps of TOM core with Tom 20 in position P_1 at 6.7 Å and position P_2 at 6.6 Å resolution (see also Fig. S7).

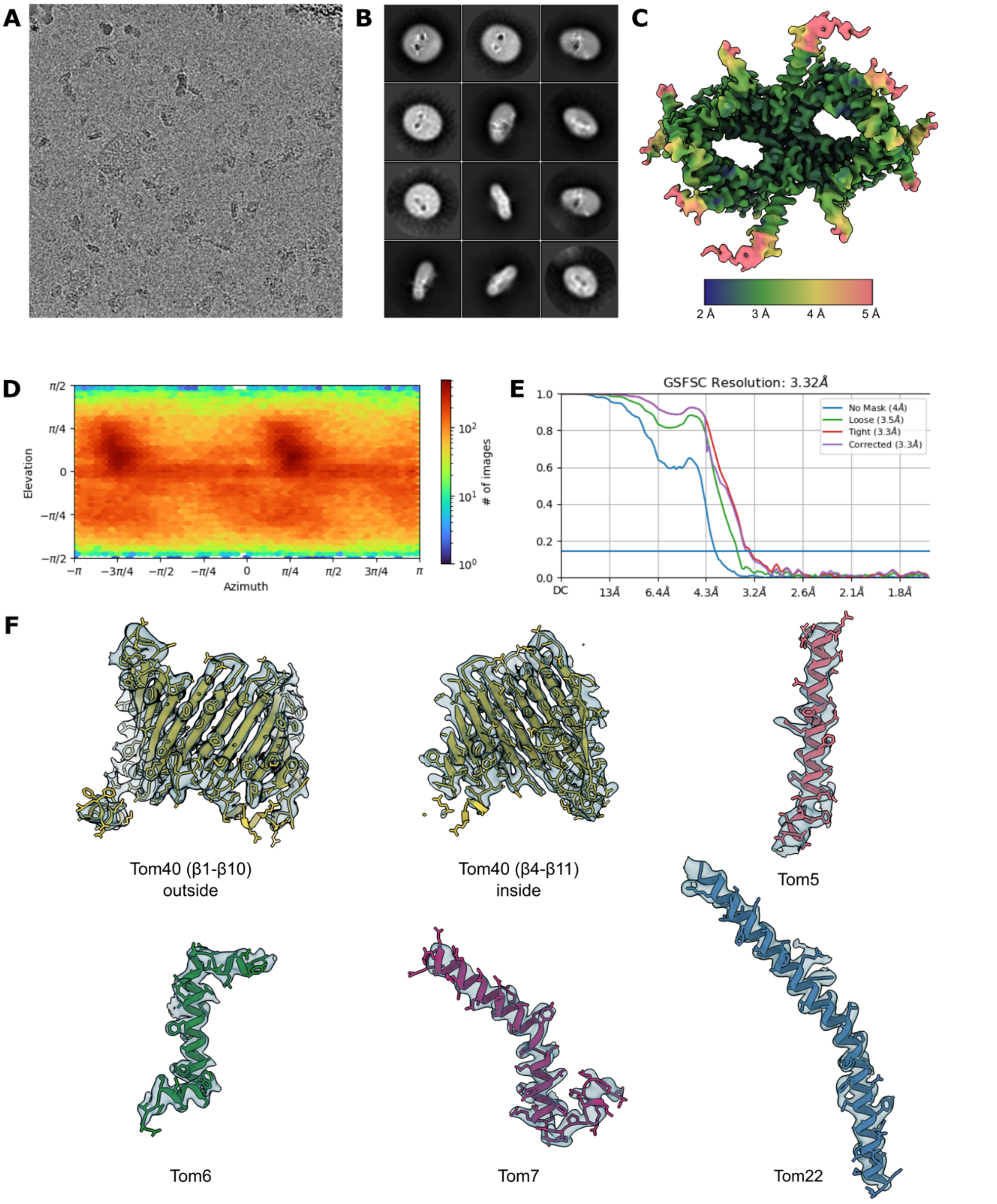

Fig. S3.

Single-particle processing of the TOM core complex. (**A**) Representative electron micrograph from the cryoEM data collection. (**B**) Representative 2D class averages of TOM complex. (**C**) Final map colored according to local resolution as estimated by cryoSPARC. (**D**) Heat map of particle distribution in the final reconstruction as measured in cryoSPARC. (**E**) Fourier shell correlation of final local refinement and local resolution estimate carried out in cryoSPARC. (**F**) The individual subunits of the TOM core complex indicate the high map quality.

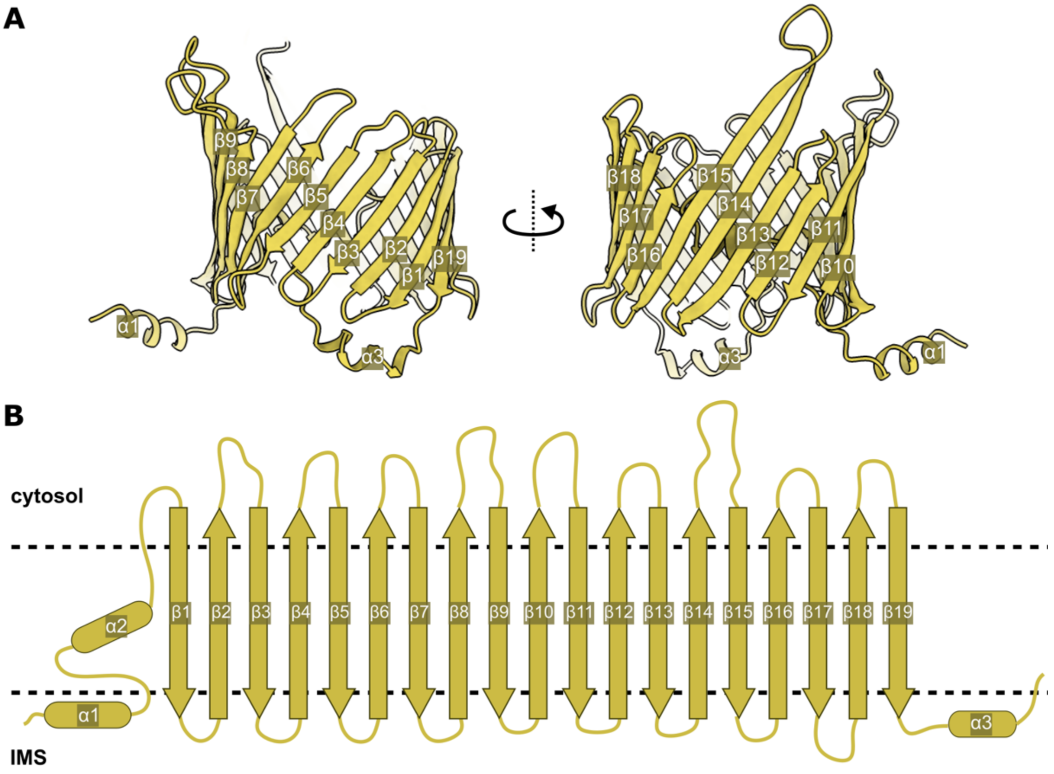

Fig. S4.

The Tom40 translocation pore. (**A**) Atomic model of Tom40 with numbered β-strands. (**B**) Schematic diagram of Tom40 secondary structure.

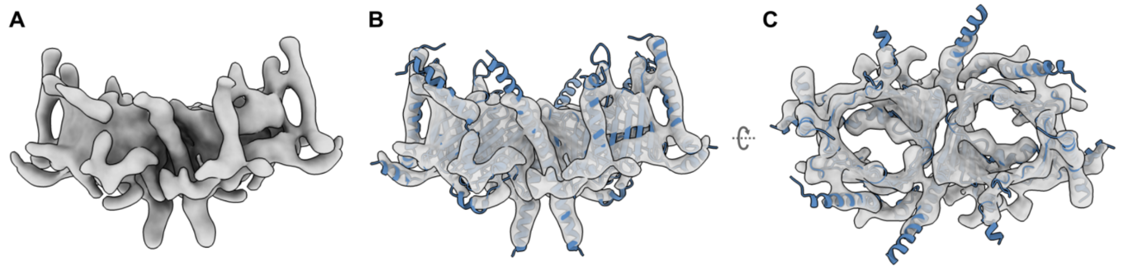

Fig. S5.

Superposition of the NcTOM core model (PDB 8B4I, blue) derived from our present 3.32 Å map, on our previous 6.8 Å map (EMDB-3761) (**A**), as seen from the side (**B**) and from the cytosol (**C**).

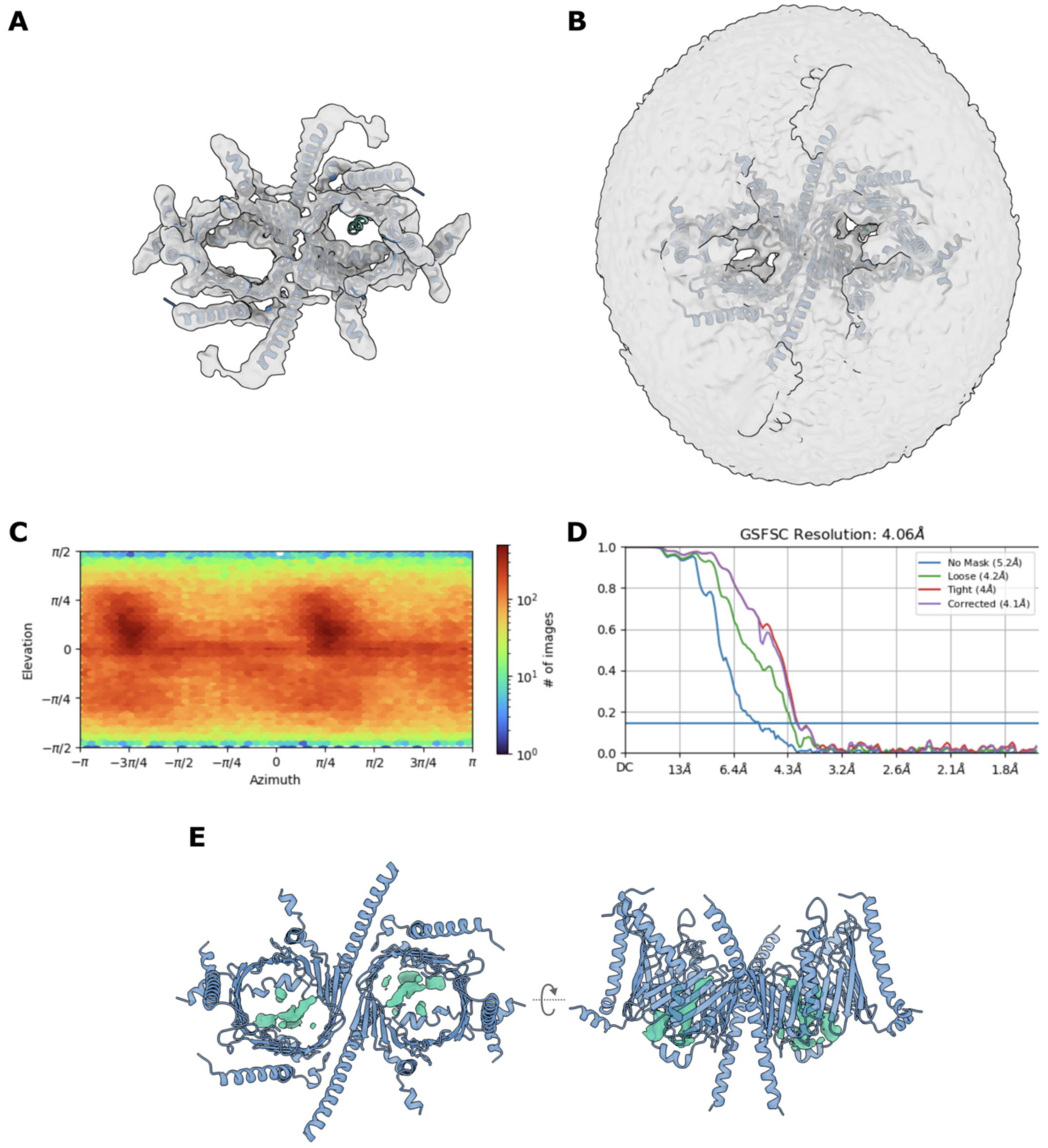

Fig. S6.

Processing of TOM core complex with bound presequence. Map obtained by non-uniform refinement with limited alignment resolution of TOM core particles with C2 symmetry applied. Superposition of 4 Å map with the TOM core model and rigid-body-fitted pALDH inside one pore at (**A**) high and (**B**) low-density threshold. (**C**) Heat map of particle distribution in the final reconstruction measured in cryoSPARC. (**D**) Fourier shell correlation of final local refinement and local resolution estimate from cryoSPARC. (**E**) Difference map showing the presequence density (green) superposed on the TOM core model (blue) as seen from the cytosol (left) or from the side.

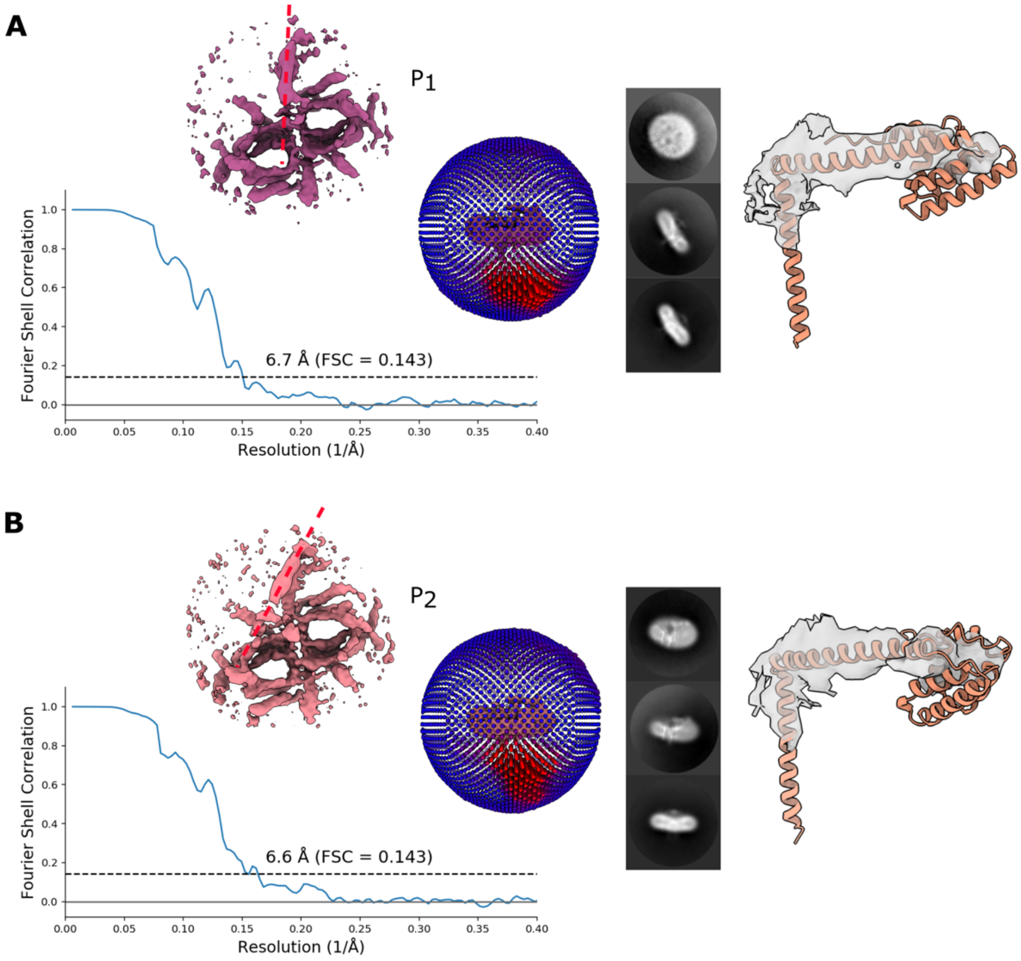

Fig. S7.

Maps of NcTOM holo complex with Tom 20 in position P_1 (**A**) or position P_2 (**B**). Dashed red lines indicate the different orientation of Tom20 in two populations of TOM holo particles. Each figure shows representative 2D averages of the final particles in each map, as well as the particle distribution in the final reconstruction, the Fourier shell correlation of the final refinement measured in Relion-4.0, and the density fit of Tom20 in the given conformation.

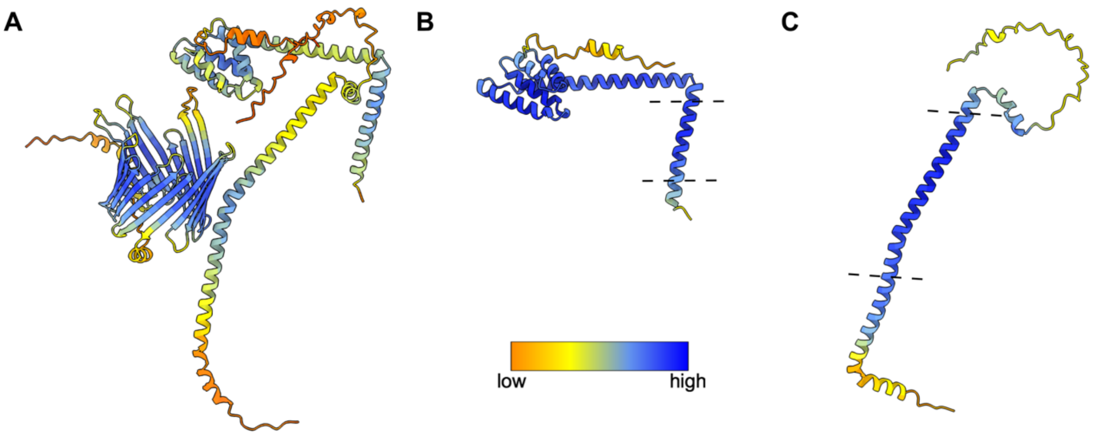

Fig. S8.

AlphaFold predictions of key TOM subunits colored by estimated per-residue confidence. Blue indicates high confidence and orange low confidence. (**A**) AlphaFold-Multimer prediction model of the $Tom20_1Tom22_1Tom40_1$ subcomplex. (**B**) and (**C**) show the monomer predictions of Tom20 and Tom22 that were used for rigid-body-fitting. Dashed lines indicate the likely position of the outer mitochondrial membrane, based on the hydrophobicity of modelled transmembrane helices.

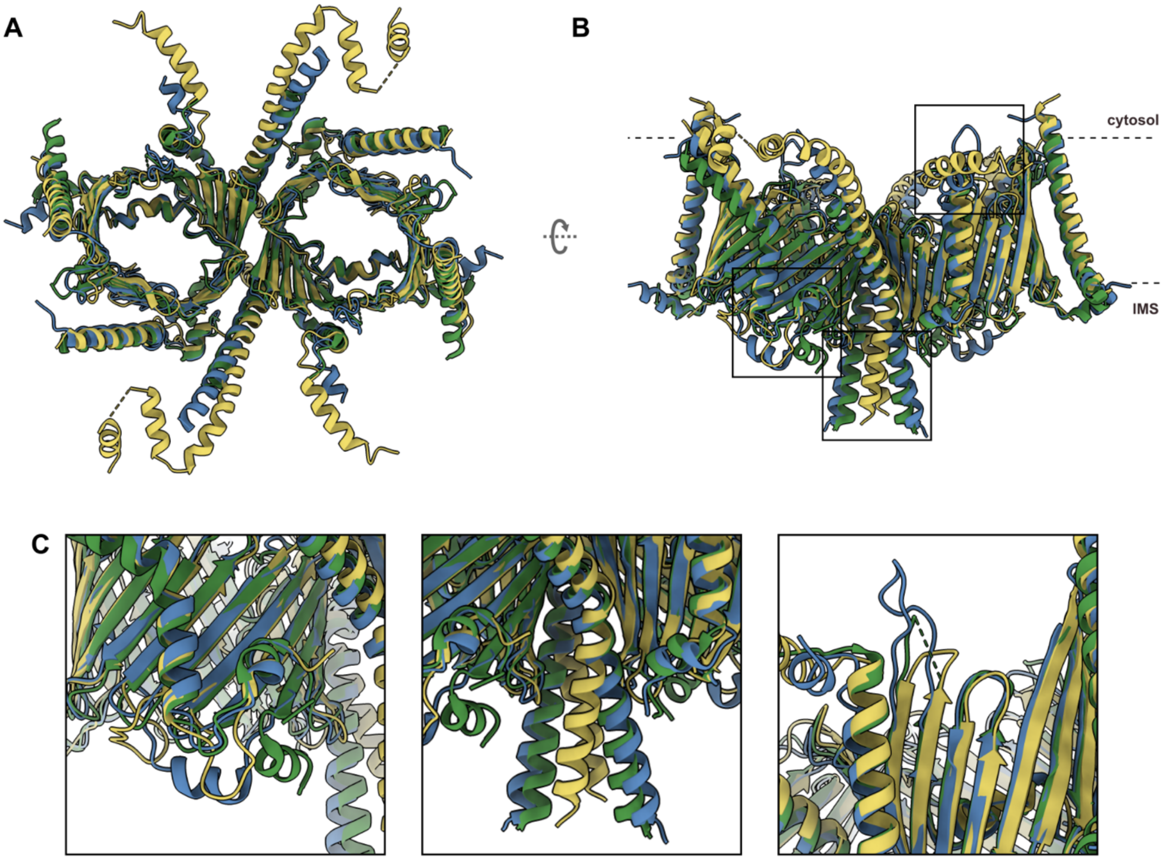

Fig. S9.

Comparison of atomic models of the TOM core complex from human, yeast and *N. crassa* mitochondria. Human TOM is shown in yellow (PDB 7CP9), yeast TOM in green (PDB 6UCU) and *N. crassa* TOM in blue (PDB 8B4I). (**A**) Cytosolic view of the three models. (**B**) Side view of the models with squares indicating the regions shown in C. Dashed lines indicate the outer membrane. (**C**) Close-up of differences in Tom7, Tom22 and Tom40.

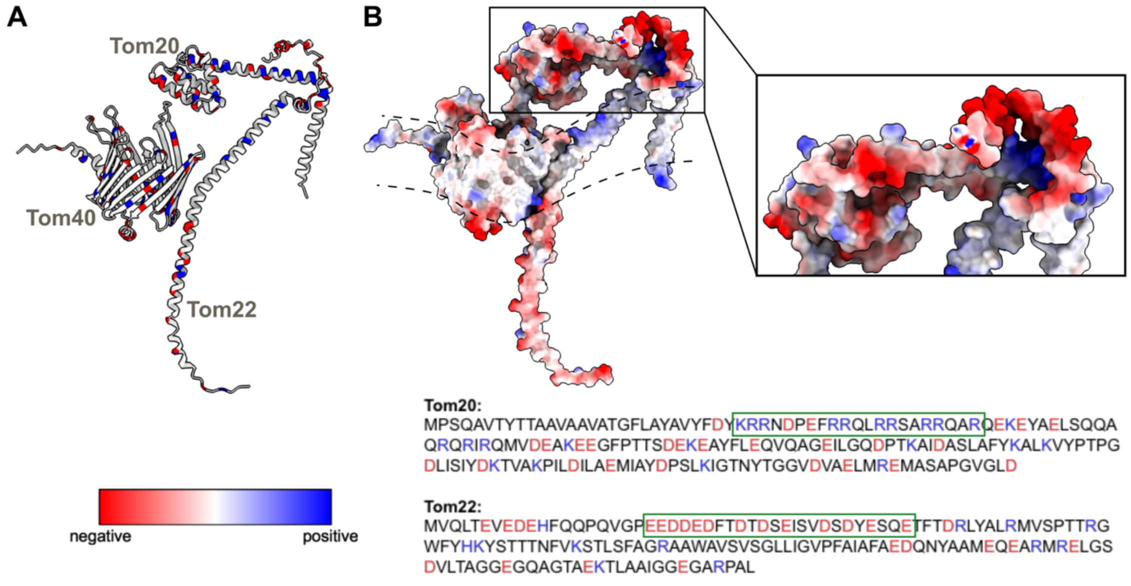

Fig. S10.

AlphaFold prediction of the $Tom20_1Tom22_1Tom40_1$ subcomplex colored by electrostatic potential. Red and blue indicate negatively and positively charged regions, respectively. (**A**) Cartoon representation with highlighted charged regions. (**B**) Close-up of the predicted docking site of Tom20 on Tom22. The N-Terminal of Tom22 is cut transversally to show the region of interest. The likely position of the outer membrane is indicated by dashed lines. Sequences of Tom20 and Tom22 are shown, color-coded by residue charge. Green boxes highlight the regions of charge complementarity in Tom20 and Tom22 that we propose holds the two subunits together by electrostatic interactions.

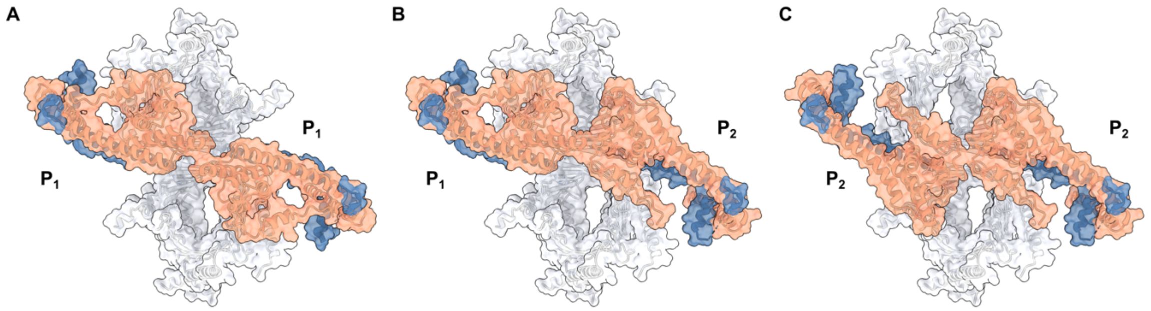

Fig. S11.

Attempts to fit two copies of Tom20 as rigid bodies into our TOM core dimer model in its two different positions: (**A**) $P_1 + P_1$, (**B**) $P_1 + P_2$ and (**C**) $P_2 + P_2$. The receptor domains of the fitted Tom20 clash in the cytosol in both conformations, making it unlikely that they can coexist in one dimer.

Table S1. Predicted and experimental mass related to the TOM holo complex, subcomplexes and subunits. The predicted masses were calculated using the ExPASy tool. The value for the unidentified sT = 5.975 kD was taken from the average of the mass of Tom5, Tom6 and Tom7.

Protein	Predicted Mass (kDa)	LILBID Mass (kDa)
Tom5	5.402	5.657
Tom7	6.061	6.231
Tom6	6.463	6.550
Tom22-6His	17.639	17.901
Tom20	20.228	20.099
Tom40	38.150	37.925
Tom70	69.340	69.363
$Tom20_2$	40.456	39.785
sT_1Tom40_1	44.125	44.020
sT_2Tom40_1	50.101	53.483
$Tom22_1Tom40_1$	54.966	55.553
$Tom20_2Tom22_1$	57.272	57.623
$sT_1Tom22_1Tom40_1$	60.941	62.650
$sT_2Tom22_1Tom40_1$	66.917	68.859
$Tom20_1Tom22_1Tom40_1$	75.194	75.692
sT_2Tom70_1	81.291	81.980
$sT_1Tom20_1Tom22_1Tom40_1$	81.169	82.857
$Tom22_1Tom70_1$	86.156	86.453
$sT_2Tom20_1Tom22_1Tom40_1$	87.145	87.762
$Tom20_1Tom70_1$	89.568	89.496
$Tom40_1Tom70_1$	107.490	107.005

Table S2. CryoEM data collection, refinement and validation of the NcTOM core complex.

	TOM core	Preprotein bound TOM core	TOM holo P_1	TOM holo P_2
**Data collection and processing**				
Magnification	105kx	105kx	105kx	105kx
Voltage (kV)	300	300	300	300
Electron exposure	55 e-/A2	55 e-/A2	55 e-/A2	55 e-/A2
Defocus Range (μm)	-1.2 to - 3.0	-1.2 to - 3.0	-1.2 to - 3.0	-1.2 to - 3.0
Pixel size (Å)	0.837	0.837	0.837	0.837
Symmetry imposed	C2	C2	C1	C1
Initial particles	1,499,000	1,499,000	1,499,000	1,499,000
Final particles	304,506	304,506	119,697	141,007
Map resolution (Å)	3.32	4.02	6.69	6.56
FSC Threshold	0.143	0.143	0.143	0.143
**Refinement**				
Initial model used	AlphaFold-Multimer			
Model resolution	3.24			
FSC Threshold	0.143			
Map sharpening B factor (Å2)	-80			
**Model composition**				
Nonhydrogen atoms	8,250			
Protein residues	1,026			
Ligands	9			
**R. m. s. deviations**				
Bond lengths (Å)	0.004			
Bond angles (˚)	0.571			
**Validation**				
MolProbity score	1.17			
Clashscore	3.81			
Poor rotamers (%)	0.00			
Ramachandran plot				
Favored (%)	98.51			
Allowed (%)	1.49			
Disallowed (%)	0.00			

Movie S1. Morph of TOM core + Tom20 models changing between positions P_1 and P_2. In P_1, Tom20 (orange) is in a central position, docked on Tom22 (blue), between the two pores of the dimer (gray). In P_2, Tom20 approaches the pore close to Tom6.
